# Prevalence of *Yersinia pestis* among rodents captured in a semi-arid tropical ecosystem of south-western Zimbabwe

**DOI:** 10.1515/biol-2022-0359

**Published:** 2022-09-03

**Authors:** Annabel Banda, Edson Gandiwa, Never Muboko, Victor K. Muposhi

**Affiliations:** Department of Wildlife Ecology and Conservation, Chinhoyi University of Technology, Private Bag 7724, Chinhoyi, Zimbabwe; Department of Crop Science, Gwanda State University, P.O. Box 30, Filabusi, Zimbabwe; Department of Wildlife and Aquatic Resources, Botswana University of Agriculture and Natural Resources, Gaborone, Botswana

**Keywords:** plague prevalence, rodents, species diversity, trapping success

## Abstract

This study assessed the prevalence of plague bacterium (*Yersinia pestis*) among rodents captured in Umzingwane and Nkayi districts, south-western Zimbabwe. A total of 44 rodents were captured on three consecutive days per trapping session in the study sites using a removal trapping method in April 2018. Captured rodents were euthanized, and blood samples were collected. The Giemsa stain method was used to detect plague bacteria. The trapping success was not significantly different (*χ*² = 1.50, df = 1, *P* = 0.221), 8.5% for the Nkayi district, while in the Umzingwane district, it was 8%. Overall, only one rodent species, i.e., *Mastomys natalensis*, tested positive for *Y. pestis* in the Umzingwane district, thus yielding a prevalence rate of 2.3% for the entire study area. This was the most important finding of a *Y. pestis*-positive rodent in a non-endemic wild area in the Umzingwane district. These results point to a low prevalence of *Y. pestis* in the study area and the importance of an active plague disease surveillance and monitoring system.

## 1 Introduction

Plague, a disease caused by the bacilli bacterium, *Yersinia pestis*, primarily affects rodents, mainly transmitted from one host to another through bites of infective fleas [1]. However, there are alternative transmission routes, such as inhalation and direct contact, which do not normally play a role in the maintenance of plague bacterium in the animal reservoirs [2]. Fleas are generalists in their feeding behavior; thus, in the absence of rodents, they seek alternative hosts, such as wild and domestic animals, as well as people [3]. Thus, some flea species, for instance, *Xenopsylla brasiliensis*, are implicated as effective plague transmitters [4]. Alternatively, humans can acquire plague by handling infected rodents or by droplet infection, although humans do not play a role in the long-term survival of the plague bacterium [2]. In most cases, a plague outbreak causes a great population decrease in rodents of some species within their local and established ranges (Barnes, 1993, cited in Thiagarajan et al. [5]). However, some rodent hosts act as reservoirs of the disease in the enzootic environment allowing low levels of the pathogen circulation [3].

The first cases of human plague in Zimbabwe were recorded in 1974. The cases showed a complicated combination of plagues [6]. From then up to 1985, there were 89 cases noted, of which 23 deaths occurred [7]. Subsequent plague human cases were recorded in 1994 and 1997, where the highest cases of plague were noted, with about 400 cases and 30 deaths confirmed in Nkayi and Lupane districts, Matabeleland North [7,8]. In 2012 (latest information), plague was detected in Zimbabwe, but there was no full report of the disease [6]. Unavailability of active plague surveillance may have adverse repercussions, as an epidemic can occur undetected [9]. Plague foci are dynamic and keep on emerging and re-emerging [10], hence the importance of research to establish any potential new plague foci.

Plague bacterium can be hosted by a number of rodents. It was detected in multimammate mouse (*Mastomys natalensis*) and *Gerbilliscus* species in Tanzania [11]. Furthermore, small mammals, such as rabbits (*Sylvilagus floridanus*), marmots (*Marmota*), and chipmunks (*Eutamias* spp.), were shown to maintain plague in the wilderness [3]. Surveying diseases among free ranging wild animal populations may provide an early warning system for the presence of a disease. There is generally less active plague surveillance in Zimbabwe [4], except the few publications on rodents and fleas which largely focus on rodent and flea species diversity [12,13]. Therefore, this study aims to fill the gap in knowledge on plague prevalence in Zimbabwe by assessing the prevalence of plague bacteria among rodent species in Nkayi and Umzingwane districts. These two districts are in the same agro-ecological zone but differ in their plague occurrence: one is plague endemic (Nkayi district) while the other is plague non-endemic (Umzingwane district) [6,7].

## 2 Materials and methods

**Figure 1:**
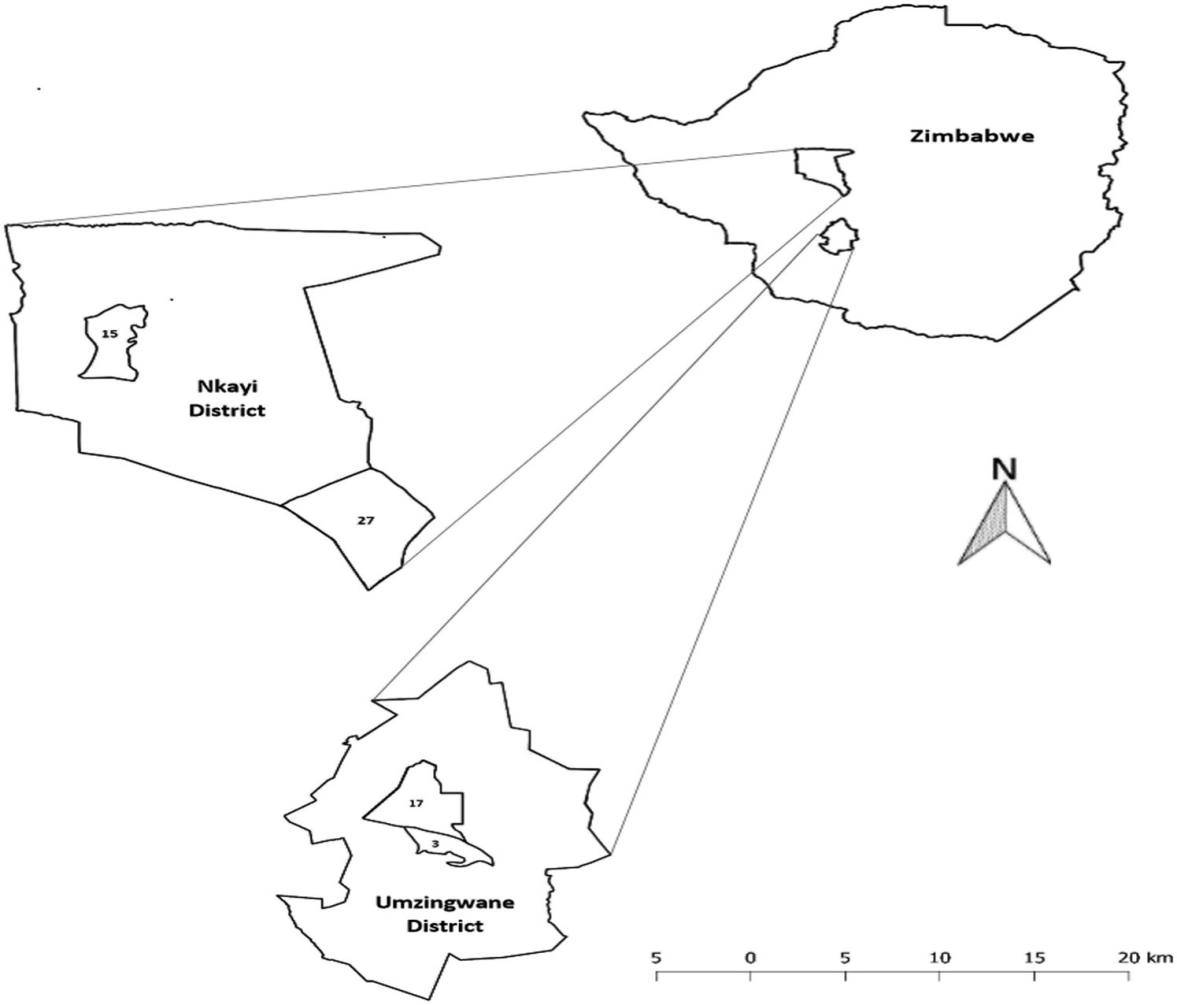
Location of Nkayi and Umzingwane Districts in south-western Zimbabwe. Notes: 15, 27, 3, and 17 represent Monki, Mathoba, Nhlekiyane, and Crocodile wards, respectively.

The study was conducted in south-western Zimbabwe in Nkayi and Umzingwane districts’ located in natural region IV (Figure 1). Nkayi district has deep Kalahari sands occupying 60% of the area whereas Umzingwane district’s soils are derived from granite rocks being coarse, sandy, and low in fertility [14]. The most common type of vegetation in Nkayi district is broad leafed woodlands, teak (*Baikiaea plurijuga*), and *Brachystegia* spp. [15]. Umzingwane district is characterized by three types of vegetation, which are bushveld, mainly covered with *Acacia* ranging between 1 and 5 m high, wooded grassland, and woodland covered by *Terminalia* and *Combretum* genus trees. The grasslands are the main source of grazing land [14].

In the Nkayi district, most of the local rural community members are engaged in extensive livestock production and cultivation of some drought-tolerant crops, such as sorghum (*Sorghum vulgare*), finger millet (*Eleusine coracana*) and pearl millet (*Pennisetum glaucum*). However, farmers do sometimes grow some short-season maize (*Zea mays*) varieties. Nkayi district’s population was reported to be 109,135 people, while the Umzingwane district had 62,990 people in 2012 [16].

This study followed a quasi-experimental design comprising two strata, i.e., districts and then two villages in each district. In the Nkayi district, the two villages studied were Mathoba and Monki villages while in the Umzingwane district were Crocodile and Nhlekiyane villages. All the trapping procedures and further processing of rodents were carried out following Dennis et al. [7], except that traps were placed 10 m apart.

Rodent trapping was conducted in April 2018 by first observing rodent activity, such as maize cob consumption and/or clearly constructed tracks and warrens. Rodents were captured in villages, i.e., bushes habitats using 15 Sherman live traps placed about 10 m apart in transects [17]. Three transects were placed in uncultivated places near fields in each study village. On the first day of sampling, traps were set late afternoon and inspected the following morning between 6 am and 7 am before it was too hot to reduce stress in captured rodents. Traps were left open for three consecutive days per village, i.e., 45 traps.

Productive traps were replaced in the morning following Kimaro et al. [17]. Traps with captures were taken to a central processing point in each study village, in an open area, for euthanization of rodents using chloroform, species identification and blood sample collection. Rodents were identified according to the illustrations and descriptions by du Plessis et al. [18]. Rodents identified were confirmed with the Natural History Museum, Bulawayo, Zimbabwe. Rodents’ cadavers were buried in a pit of about 50 cm deep.

After about 2–3 min after introducing the rodent to chloroform, blood was collected from the euthanized rodent using a hypodermic needle with a connected syringe. Blood was collected from the heart blood on which blood smears were made. Slides with Giemsa stain were examined at ×1,000 for 5–10 min, searching for bipolar coccobacilli [19] in the Wildlife Laboratory at Chinhoyi University of Technology, Chinhoyi, Zimbabwe.

### 2.1 Data analysis

Trapping success was calculated as the number of animals caught divided by the trapping period divided by the number of traps set per period multiplied by 100 (7). The Chi-square (*χ*²) test of independence or cross-tabulation was used to determine if there were differences among the trapping success in STATISTICA version 10 for Windows [20]. Diversity of rodents in each district was calculated following the Shannon–Wiener (*H*′) index measure of diversity [21]. The prevalence of *Y. pestis* in rodents’ blood samples was calculated as the number of *Y. pestis*-positive rodent individuals divided by the total number of rodents examined multiplied by 100.

## 3 Results

A total of 44 rodents were captured in the two study districts, i.e., Nkayi (*H*′ = 0.89) and Umzingwane (*H*′ = 0.62). There was no significant difference in the trapping success between the study districts, i.e., Nkayi (8.5%) and Umzingwane (8%) (Table 1) (*χ*² = 1.50, df = 1, *P* = 0.221). Twenty-three (23) rodents were caught in the Nkayi district and 21 in the Umzingwane district, i.e., 17 *M. natalensis* (Smith, 1834), 6 *Gerbilliscus brantsi* (Smith, 1836), 4 *Gerbilliscus leucogaster* (Peters, 1852), and 17 *Saccostomys campestris* (Peters, 1846). Only one rodent, i.e., *M. natalensis*, caught in the Umzingwane district tested positive for the plague bacterial, indicating an overall prevalence of plague of 2.4% in the study area (Table 2).

**Table 1:** Rodents trapping success in the study area

Variable	Nkayi district		Umzingwane district	
	Mathoba	Monki	Crocodile	Nhlekiyane
No. rodents per site	10	13	13	8
Trapping success (%)	7	10	10	6
Mean trapping success (%)	8.50		8.00	

**Table 2:** Rodents captured species diversity and prevalence of bacterial plague in the study area

Rodent species	Nkayi district			Umzingwane district			Overall prevalence (%)
	# of rodents captured	# of rodents that are positive for *Y. pestis*	Prevalence (%)	# of rodents captured	# of rodents that positive for *Y. pestis*	Prevalence (%)	
*M. natalensis*	0		0	17	1	5.9	5.9
*S. campestris*	4	0	0	2	0	0	0
*G. brantsi*	4	0	0	2	0	0	0
*G. leucogaster*	15	0	0	0	0	0	0
Species diversity (*H′*)	0.89	—	—	0.62	—	—	—
Total/average (%)	23	0	0	21	1	4.8	2.4

Note: – denotes not applicable; # – denotes number.

## 4 Discussion

This study is the first to report the presence of plague bacteria among rodents in Umzingwane district, even though a low prevalence of plague disease exists in the study area. Elsewhere, plague bacteria were reported as being difficult to detect in rodents and fleas associated with prairie dog colonies (*Cynomys*) at Thunder Basin National Grassland in Wyoming, USA [5]. Thus, it was suggested that where possible plague determination investigations could be conducted in places where there are noticeable rodent die-offs [5]. Since time immemorial *M. natalensis* was observed not to easily succumb to plague bacteria, thus termed an enzootic host [11,22]. Thus, some of the caught species may not be ideal host for *Y. pestis*, for instance *T. leucogaster* was observed to easily succumb to *Y. pestis* closely related bacilli *Y. pestis* tuberculosis subspecies *pestis* [23].

Since no die-offs were observed during the sampling time, it may imply that an inter-epizootic period could have existed during which *Y. pestis* cannot be recovered from fleas, rodents, or any other host [1]. Instead, it was proposed in Iran and Madagascar that the bacteria could remain in existence in the soil. However, one study recommended that the transmission route by exposure of susceptible mice to *Y. pestis*-contaminated soil seems doubtful under natural conditions because the infectious period was short-lived and the transmission efficiency was low [24]. Thus, during plague quiescent times, it is likely that detecting *Y. pestis* is by mere chance.

In northern Tanzania, 517 wild, peri-domestic, and small commensal mammals, including rodents and wild carnivores, were captured, but only three tested positive for *Y. pestis*. This is an indication for the need for large samples to fully get a good picture of the infection status of a rodent population [11]. There can, however, be possibilities that *Y. pestis* is concentrated on certain rodent body organs as was reported in a study in Mongolia, where *Y. pestis* was detected in spleen samples, while liver samples from rodents tested negative using Polymerase Chain Reaction [25]. This therefore points to the importance of a large sample associated with diversified samples from the rodent body parts to enhance the chances of detection of the representative infection status of populations.

## 5 Conclusion

The study provides the first evidence of plague bacteria infection in a rodent species in Umzingwane district, a non-endemic region for the plague disease. It is recommended that future studies on plague bacteria assessment should involve a larger sample both for rodent populations and areas covering endemic and non-endemic regions, cover a longer sampling timeframe, and take samples from other body parts of rodents (liver and spleen) for testing beyond the blood.
